# Electron Beam-Induced Immobilization of Laccase on Porous Supports for Waste Water Treatment Applications

**DOI:** 10.3390/molecules190811860

**Published:** 2014-08-08

**Authors:** Elham Jahangiri, Senta Reichelt, Isabell Thomas, Kristin Hausmann, Dietmar Schlosser, Agnes Schulze

**Affiliations:** 1 Helmholtz Centre for Environmental Research, Permoserstr. 15, D-04318 Leipzig, Germany; E-Mails: elham.jahangiri@ufz.de (E.J.); dietmar.schlosser@ufz.de (D.S.); 2 Leibniz Institute of Surface Modification, Permoserstr. 15, D-04318 Leipzig, Germany; E-Mails: senta.reichelt@iom-leipzig.de (S.R.); isabell.thomas@iom-leipzig.de (I.T.); kristin.hausmann@iom-leipzig.de (K.H.)

**Keywords:** cryogels, membranes, laccase immobilization, electron beam irradiation, bioreactor, degradation of pollutants, redox mediator immobilization

## Abstract

The versatile oxidase enzyme laccase was immobilized on porous supports such as polymer membranes and cryogels with a view of using such biocatalysts in bioreactors aiming at the degradation of environmental pollutants in wastewater. Besides a large surface area for supporting the biocatalyst, the aforementioned porous systems also offer the possibility for simultaneous filtration applications in wastewater treatment. Herein a “green” water-based, initiator-free, and straightforward route to highly reactive membrane and cryogel-based bioreactors is presented, where laccase was immobilized onto the porous polymer supports using a water-based electron beam-initiated grafting reaction. In a second approach, the laccase redox mediators 2,2'-azino-bis(3-ethylbenzothiazoline-6-sulphonic acid) (ABTS) and syringaldehyde were cross-linked instead of the enzyme via electron irradiation in a frozen aqueous poly(acrylate) mixture in a one pot set-up, yielding a mechanical stable macroporous cryogel with interconnected pores ranging from 10 to 50 µm in size. The membranes as well as the cryogels were characterized regarding their morphology, chemical composition, and catalytic activity. The reactivity towards waste- water pollutants was demonstrated by the degradation of the model compound bisphenol A (BPA). Both membrane- and cryogel-immobilized laccase remained highly active after electron beam irradiation. Apparent specific BPA removal rates were higher for cryogel- than for membrane-immobilized and free laccase, whereas membrane-immobilized laccase was more stable with respect to maintenance of enzymatic activity and prevention of enzyme leakage from the carrier than cryogel-immobilized laccase. Cryogel-immobilized redox mediators remained functional in accelerating the laccase-catalyzed BPA degradation, and especially ABTS was found to act more efficiently in immobilized than in freely dissolved state.

## 1. Introduction

An emerging issue of the 21st century is related to micro-pollutants increasingly detected in waters. Such compounds are typically found in the ng/L to the lower µg/L range and are often not or not sufficiently removed in conventional wastewater treatment plants. Micro-pollutants include diverse compounds of various origins and uses (e.g., industrial chemicals, pesticides, pharmaceuticals and personal care products), and may be emitted from diffuse as well as point sources [1,2,3]. For instance, anything from half to almost all of a pharmaceutical drug taken by a patient can pass through the body without being absorbed or metabolized and the original form of a drug can also be regenerated from excreted metabolites, together with incomplete elimination in wastewater treatment plants resulting in chronic contamination of the environment by drug residues [4,5]. Major concerns about micro-pollutants in the environment are related to potentially hazardous, undesirable biological activities such as, e.g., endocrine disruption [1,6].

To eliminate critical contaminants from water, recent trends favor environmentally friendly technologies. The use of enzymes is environmentally benign, efficient, and more selective compared to chemical catalysts. For instance, enzymes enable milder reaction conditions, and may lead to higher reaction rates. The oxidoreductase laccase, which is prominent in fungi but also known from plants, bacteria, and insects, has been intensively studied for the purpose of oxidation-mediated degradation of pollutants, and to elucidate the involved oxidation mechanisms [7,8]. Laccase redox mediators, small molecules of synthetic or natural origin representing laccase substrates, are known to speed up the oxidation rates of various environmental pollutants and to expand laccase substrate ranges to compounds that cannot directly be attacked by laccases. Hence, such compounds are very attractive for environmental biotechnology, and have gained much attention. However, there are also major drawbacks related to certain laccase redox mediators such as sometimes high costs, losses caused by irreversible oxidation/degradation and hence comparatively high concentrations needed for efficient catalysis, losses from reaction systems in case of continuous operation, and sometimes toxic by-products [8,9]. 

The use of free enzymes is limited due to their labile nature, which makes a reuse difficult. In contrast, immobilized enzymes may offer several advantages such as, e.g., an improved thermal and operational stability, an enhanced activity resulting from the stabilization of a hyperactivated form of the enzyme, and improvements with respect to enzyme specificity or selectivity [10,11,12]. By immobilizing enzymes on a support the system offers furthermore the advantage of easy separation of the enzyme from the reaction mixture while the product will not be contaminated by the biocatalyst [13]. Laccase-based degradation processes can be efficiently applied using biocatalytic membrane reactors. These membrane reactors combine two tasks as the membrane serves as a support material for the biocatalyst and simultaneously separates the products by size exclusion. Different examples for immobilization of enzymes on polymer supports such as membranes, by adsorption, covalent coupling, cross-linking, and incorporation in the polymer bulk have been discussed previously [14,15,16,17,18,19]. Previous studies focused on the immobilization of laccase on polyethersulfone membranes by surface adsorption [20], or by covalently coupling a pre-functionalized hydrophilic polyvinylidene membrane (using hydrazine) with an aldehyde-derivatized laccase [21]. Furthermore, covalently coupling of laccase on a polyamide membrane was performed using glutaraldehyde (toxic) as coupling agent [22], or by immobilization (by surface adsorption or by glutaraldehyde coupling) of laccase onto TiO_2_ nanoparticles followed by incorporation of these particles into the membrane polymer (mixing with the polymer solution used for membrane preparation) [23]. In the context of biocatalyst immobilization for pollutant degradation, we are not aware of attempts to immobilize laccase redox mediators (maybe instead of or along with the catalyzing enzyme). Provided that stable binding of redox mediators to suitable carriers together with the maintenance of their functionality could be achieved, this would provide a very attractive approach with the potential to circumvent known drawbacks of redox mediators related to losses from the reaction system as mentioned before.

Recently, we developed a new approach to covalently immobilize the enzyme trypsin on different commercial membranes (pore sizes: 0.22–0.45 µm) via electron beam (E-Beam) irradiation [24]. This approach combines surface activation of the matrix polymer and simultaneous immobilization of the enzyme by use of low-energy E-Beam in an aqueous system. The procedure neither requires any preceding surface functionalization, no prior functionalization of the enzyme, nor the use of catalysts or other toxic reagents.

In the course of this publication we furthermore introduce a second in-situ E-Beam-based approach to immobilize enzymes using macroporous polymeric cryogels (MPC) as carrier. MPCs are characterized by a three-dimensional network of interconnected pores ranging from 1–100 µm in size and a high porosity of about 90%. Usually, a solution of organic precursors and initiators is dissolved in water, frozen, if appropriate initiated, and stored at temperatures about −20 °C to −30 °C during reaction [25]. By using high-energy electron radiation cryogels can be synthesized without additional initiators or cross-linkers within 10 min (excl. freezing time) as larger bulk materials allowing furthermore the simultaneous incorporation of macromolecules in a one-step process [26,27,28].

Generally, the immobilization of biomolecules by high-energy radiation is combined with limitations. Radiolytic inactivation appears due to the fragmentation or the modification of amino acid side chains [29,30]. Several groups have studied the mechanism of radiolytic inactivation of proteins. Differences in the degradation behavior of proteins were observed in dependence on their physical state (aqueous solution, frozen aqueous solution or lyophilized) [31]. It was demonstrated that the radiation damage was decreased at lower temperatures [31,32]. In preliminary studies with trypsin as incorporated enzyme we could already prove that E-Beam at room temperature led to inactive enzymes, whereas the activity remains very high after irradiation at −15 °C [33].

**Figure 1:**
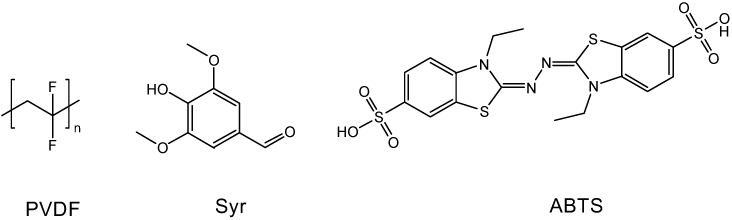
Chemical structures of the membrane polymer PVDF, and the redox mediators Syr and ABTS.

In this study a commercially available laccase from the white-rot *Trametes versicolor* was used for immobilization on the two porous polymer systems (membrane and cryogel) to prepare bioreactors using two different E-Beam-based techniques. In a second approach the two laccase redox mediators [syringealdehyde = Syr; 2,2'-azino-bis(3-ethylbenzothiazoline-6-sulphonic acid) = ABTS] (Figure 1) were immobilized instead of the enzyme laccase, in order to evaluate the possibility of circumventing known drawbacks related to the use of freely dissolved redox mediators as mentioned before. Syr is a natural phenolic compound previously reported to act as a ‘true’ redox mediator, *i.e.*, being re-reduced after oxidation by pollutants thus becoming again available for the catalytic cycle [34,35], whereas ABTS is a synthetic redox mediator partly being degraded during follow-up reactions of its laccase-oxidized form [36]. In both cases, a water-based “green” *in-situ* immobilization approach is used. For the immobilization on commercial polyvinylidene fluoride (PVDF) membranes low-energy E-Beam was applied. For cryogel synthesis we used high-energy E-Beam. One main advantage besides the abstinence of initiators/toxic cross-linkers, and the water-based approach is the evenly distribution of the enzyme/redox mediators through the entire cross-section of the porous polymer matrix. The membranes and cryogels were characterized regarding their morphology, chemical composition, and catalytic activity. The catalytic activity was investigated by using the endocrine disrupting compound bisphenol A (BPA), a widespread environmental contaminant applied in the production of epoxy resins [6,37], as a model pollutant in degradation experiments.

## 2. Results and Discussion

### 2.1. Membranes and Cryogels as Carrier Materials – Morphological and Physico-Chemical Properties

**Figure 2:**
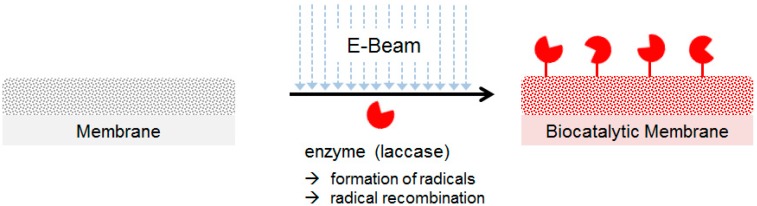
Procedure for the coupling of laccase on a polymer membrane using E-Beam irradiation.

For the immobilization of laccase on membranes we used an E-Beam-initiated grafting method. The E-Beam treatment results in the generation of a mixture of ions, excited molecules and free radicals as described for the radiolysis of water ensuring the activation of both the dissolved enzyme [24] as well as of the membranes [38,39,40]. The formed radicals/activated species can undergo various reactions, such as cross-linking or recombination reactions. This way, links between the polymer matrix and the enzyme or redox mediators can be formed (Figure 2).

Since radicals are formed at the membrane polymer surface as well as at the dissolved enzyme simultaneously, chemical bonds can be formed by recombination of adjacent radicals (e.g., a polymer radical and an enzyme radical) in a nonspecific manner. It is worth emphasizing that the use of an aqueous system is a crucial requirement since different results were obtained in the dry state as also reported in the literature [41].

This E-Beam immobilization method was compared with a common used physical adsorption immobilization method (coating without E-Beam) at the membrane surface.

**Figure 3:**
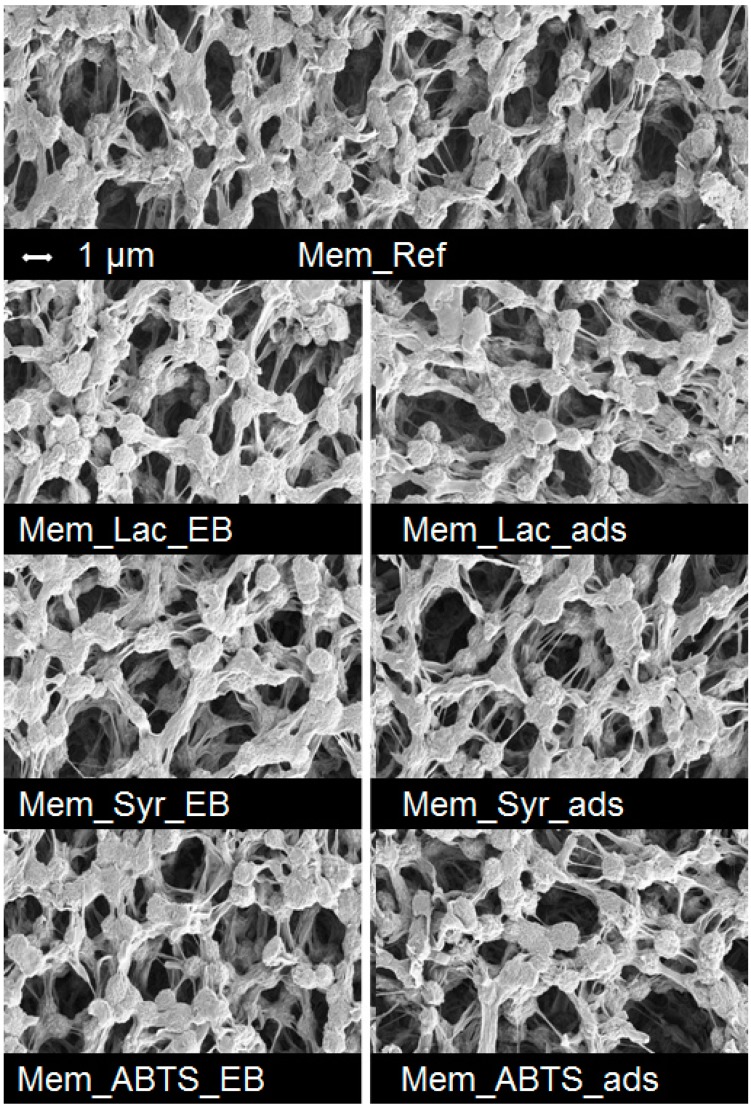
SEM images of the untreated reference membrane and of the membranes after immobilization of laccase, Syr, and ABTS using E-Beam treatment and by physical adsorption.

SEM analysis of the treated membranes indicates that no coating layer or pore blocking was obtained by laccase or by the redox mediators on the surface (Figure 3). Furthermore, the E-Beam treatment did not lead to any damages of the membrane e.g., cracks or hole formation. The PVDF membranes show a similar pore and surface structure after the different immobilization experiments using E-Beam irradiation (Figure 3, left side) compared to the non-irradiated membranes (immobilization by physical adsorption), respectively (Figure 3, right side).

The successful immobilization of the different compounds (Figure 1) on the membrane surface was confirmed by XPS analysis (Table 1).When compared to the reference membrane it is obvious that the immobilization of laccase resulted in a surface with a significantly increased content of oxygen (E-Beam: 7.1%, physical adsorption: 3.9%) and nitrogen (E-Beam: 3.1%, physical adsorption: 2.2%). Since these two atoms are not present in the membrane polymer PVDF, they can be attributed to the immobilized enzyme on the membrane surface. Furthermore, the immobilization of laccase seems to be superior when treated by E-Beam compared to physical adsorption because both oxygen and nitrogen amount are higher when treated with E-Beam.

**Table 1:** Atomic composition at the surface of the membranes after immobilization of laccase, syringaldehyde, and ABTS using E-Beam treatment and physical adsorption, respectively, as determined by XPS analysis.

	Elemental ratio (relative atom %)			
Label	F	O	N	C
**Mem_Ref**	51.0	0.8	-	48.2
**Mem_Lac_EB**	36.7	7.1	3.1	53.1
**Mem_Lac_ads**	42.9	3.9	2.2	50.1
**Mem_Syr_EB**	48.1	1.9	0.1	49.9
**Mem_Syr_ads**	48.7	1.9	-	49.5
**Mem_ABTS_EB**	50.1	1.0	0.2	48.7
**Mem_ABTS_ads**	49.0	1.0	0.2	49.9

These results could be confirmed by the BCA test for determination of the laccase amount that was immobilized to the membrane: in the case of E-Beam treatment 7.7 µg laccase/mg membrane was found, physical adsorption resulted in 6.9 µg laccase/mg membrane. Since both methods were conducted using a similar laccase concentration and similar treatment time, this can be explained with the fact that E-Beam treatment leads to covalent attachment of the enzyme to the membrane surface while laccase that was physically adsorbed to the membrane surface can be re-extracted during the washing steps after the immobilization procedure. Compared to the large enzyme molecule the two redox mediators could not be detected with the same high quantities, however, in both cases an increased amount of oxygen (syringaldehyde: 1.9%, ABTS: 1.0%) was found compared to the untreated membrane (Mem_Ref).

**Figure 4:**
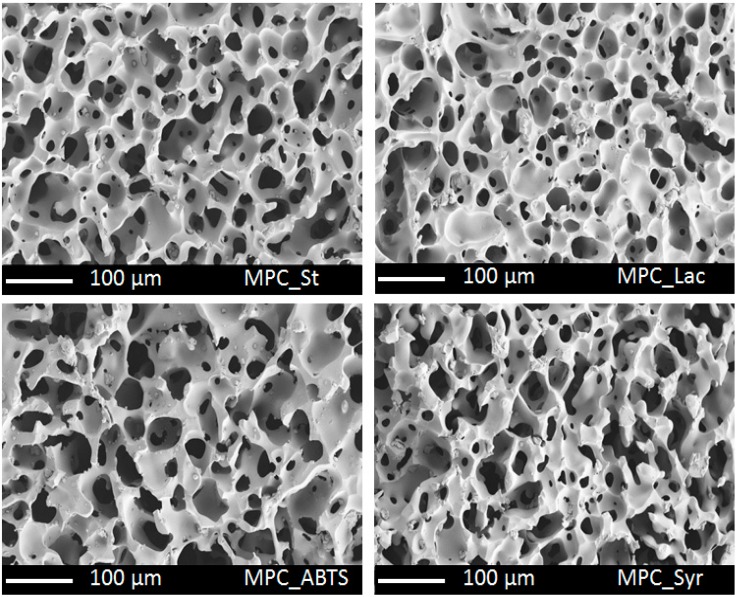
SEM images of the morphology of the standard cryogel, and of the cryogels with immobilized laccase, ABTS, syringaldehyde.

In a second approach laccase and two different redox mediators were incorporated in a porous cryogel during the cryogel synthesis, respectively. Macroporous polymeric cryogels with incorporated laccase, syringaldehyde and ABTS were successfully synthesized by E-Beam initiated cross-linking and yielded mechanical stable porous gels with a sponge-like morphology and pore sizes in the range of 50 µm (Figure 4). The irradiation time was no longer than 10 min. The crosslinking reaction leading to the macroporous material is initiated by secondary electrons which were created by a pulsed electron beam in the semi-frozen medium. As a consequence radicals or radical ions are formed which starts the reaction or form cross-linkable active sites. While the reaction mechanism of electron-initiated curing of acrylate and methacrylate bearing molecules is well understood, it needs to be investigated for enzymes or other functional molecules [42]. In a previous study we investigated the reaction mechanism of the grafting of poly(allyl amine) to polymeric monoliths by quantum chemical calculation [43]. The study revealed that poly(allyl amine) reacts with the radical species in course of an addition reaction and becomes grafted to the monolith’s surface.

The influence of the immobilized laccase and redox mediators on the morphology and thermal stability was low. The thermal degradation of the gels started at 200 °C in all cases. This may be due to the fact that the bioactive substances are not directly incorporated in the cryogel matrix but grafted to side and end chains of the cross-linked polyacrylate matrix.

**Figure 5:**
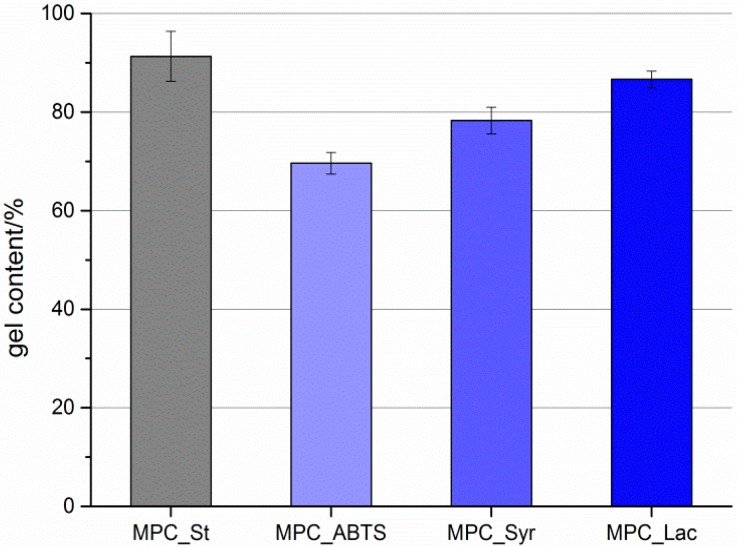
Gel content of a standard cryogel as well as with immobilized laccase, ABTS, and syringaldehyde.

The gravimetric gel content was examined from the ratio of the weight of the dry cryogel and the educt weight. As presented in Figure 5 the gel content decreased with increasing concentration of co-monomer. This might be caused by inhibition of the reaction due to the low molecular weight molecules. This effect was already described for the immobilization of different molecular weight polyethylene glycols [28]. The swelling ratio of MPC_St was in the order of 7.0 ± 0.4. The XPS analysis of the cryogel composition is hampered by the highly porous shape of the cryogels and the low sampling depth of the method (<10 nm). The examined atomic concentrations of sulfur and nitrogen for MPC_Lac and MPC_ABTS were close to the detection limit of the method (<1 atom %).

### 2.2. Activity and Stability of Membrane- and Cryogel-Based Bioreactors

Laccase could be successfully immobilized on the membranes, yielding recoveries of about 10 and 7% of the initially applied activity after immobilization using E-Beam and physical adsorption, respectively (Table 2). However, since the enzyme immobilization is accomplished by simultaneous coating with laccase solution and irradiation of the membrane (see also Section 2.1 and Figure 2) it is not expected that every enzyme molecule will be close to the membrane surface at the event of irradiation to form a covalent bond. Nevertheless, higher activity yields would likely also be achieved upon optimization of the immobilization procedures applied within the present study. Similar total laccase activities recovered after immobilization (calculated as the sum of laccase activity recovered from the washing solution and the immobilized laccase on the basis of the data shown in Table 2, respectively) of 65 (E-Beam) and 69% (physical adsorption) indicate an inactivation of the enzyme during the immobilization procedures. However, the activation loss seems not to be caused by the E-Beam treatment since the washing solutions of both experiments (immobilization by E-Beam and physical adsorption) resulted in comparable activities. Probably the contact of the enzyme with the polymer surface leads to denaturation of the enzyme to a certain extent as has been already discussed in previous studies [24]. The apparent activities of the immobilized laccase towards ABTS as a substrate corresponded to approximately 0.17 and 0.11 U/mg dry mass for irradiated and non-irradiated membranes, respectively. Together with a slightly higher recovery of laccase activity in the washing solution of the non-irradiated than in that of the irradiated membranes (Table 2), these results are in line with a more efficient binding of the enzyme upon E-Beam irradiation than by physical adsorption as already observed by XPS analysis (Table 1) and by the BCA test for determination of the immobilized laccase concentration (Section 2.1). Moreover, an apparent specific laccase activity of about 22.1 U/mg laccase deduced for E-Beam immobilized laccase on membranes (calculated from values of 0.17 U/mg membrane as above and 7.7 µg laccase/mg membrane, see Section 2.1) *vs.* an apparent specific activity of about 15.9 U/mg laccase for physical adsorbed laccase on membranes (calculated from values of 0.11 U/mg membrane as above and 6.9 µg laccase/mg membrane, see Section 2.1) points to an activity-stabilizing effect of the E-Beam irradiation. Such an effect may have been caused by a stabilization of the active conformation of the enzyme by E-Beam-induced covalent binding of the enzyme protein to the membrane surface. Similarly, activity-stabilizing effects of covalent binding have been reported for laccase immobilization via cross-linking techniques [44,45].

**Table 2:** Distribution of laccase activity within different fractions resulting from laccase immobilization on membranes by E-Beam and physical adsorption.

Fraction	Activity *(U [% recovery of activity] **)	
	Mem_Lac_EB	Mem_Lac_ads
**Primary solution**	8.85 ± 0.11 (100)	8.85 ± 0.11 (100)
**Washing solution** ***	4.88 ± 0.43 (55)	5.47 ± 0.47 (62)
**Immobilized laccase**	0.92 ± 0.02 (10)	0.59 ± 0.01 (7)

* Data represent means ± standard deviations for triplicate determinations. ** % of initial activity in the primary solution used for immobilization. *** Total laccase activity recovered from the washing steps applied after immobilization (please refer to the experimental section for details).

Laccase was also successfully immobilized in cryogels using E-Beam treatment and physical adsorption, where recoveries of about 3% and 11% of the initially applied activity for cryogels containing E-Beam-immobilized and physically adsorbed laccase, respectively (Table 3), were obtained. The cryogel-based bioreactors are synthesized in a straightforward one-pot approach. The laccase will be immobilized at the surface of the pore walls as well as within the bulk phase, and therefore, not all laccase is available at the surface of the cryogel for reaction. This explains the low recovery of the initially applied activity from cryogels (Table 3). The activity might also be reduced by radiation-induced degradation effects. However, as proven in previous studies the loss in activity is not higher than 15% [32]. Despite the relatively low amount of accessible active laccase, the high activity of the porous cryogel reactors will be presented in the following. As for the membrane immobilization, higher activity yields may be achieved by optimization of the immobilization parameters as already successfully demonstrated previously [44], e.g., by E-Beam grafting of laccase to preformed cryogels. The relatively low amount of physically adsorbed laccase is caused by the inhibition of unspecific protein adsorption due to the hydrophilic character of the cryogel. The activities of cryogel-immobilized laccase corresponded to approximately 0.013 and 0.026 U/mg dry mass for E-Beam immobilized laccase cryogels and cryogels with adsorbed laccase, respectively; thus exhibiting considerably lower enzyme loads (in terms of activity) per unit of carrier dry mass than was observed with membrane immobilization (see above). Total laccase activities recovered after cryogel immobilization (calculated as the sum of laccase activity recovered from the washing solution and the immobilized laccase on the basis of the data shown in Table 3, respectively) of 14 (E-Beam immobilized cryogels) and 65% (physical laccase adsorption, Table 3) indicate that the majority of the enzyme might be immobilized inside the polymer bulk. 

**Table 3:** Distribution of laccase activity within different fractions resulting from laccase immobilization using cryogels.

Fraction	Activity *(U [% recovery of activity] **)		
	MPC_Lac_EB	MPC_Lac_ads
**Primary solution** ***	3.58 ± 0.07 (100)	2.30 ± 0.15 (100)	
**Washing solution** ****	0.39 ± 0.05 (11)	1.25 ± 0.12 (54)	
**Immobilized laccase**	0.12 ± 0.02 (3)	0.24 ± 0.09 (11)	

* Data represent means ± standard deviations for triplicate determinations. ** % of initial activity in the primary solution used for immobilization. *** Cryogel reaction formulation for E-Beam-supported laccase immobilization, pure laccase solution in PBS for unspecific laccase adsorption; **** Total laccase activity recovered from the washing steps applied after immobilization (please refer to the experimental section for details).

**Figure 6:**
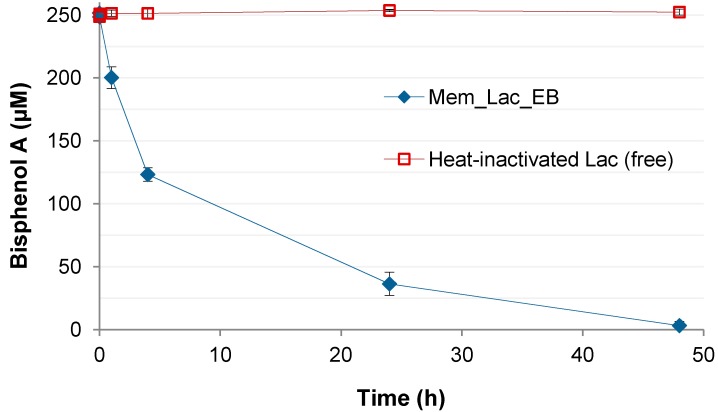
Time courses of BPA concentrations in degradation experiments employing irradiated laccase-containing membranes (Mem_Lac_EB), and in control experiments where freely suspended (non-immobilized), heat-inactivated laccase was applied (please refer to the Experimental Section for details). Data represent means ± standard deviations for triplicate experiments.

**Figure 7:**
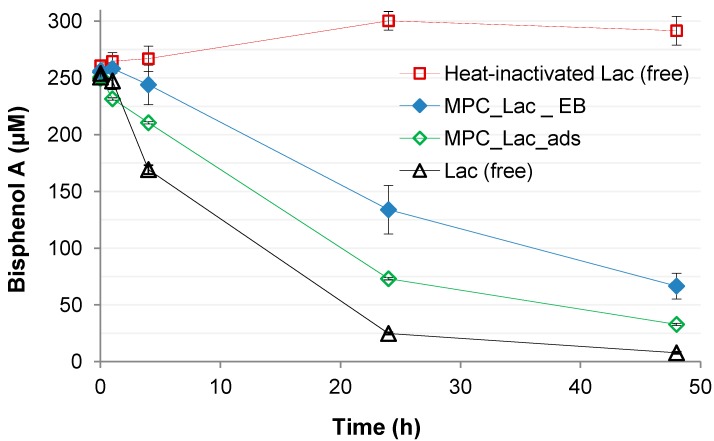
Time courses of BPA concentrations in degradation experiments with cryogels containing E-Beam-immobilized laccase (MPC_Lac_EB) and cryogels containing unspecifically adsorbed laccase (MPC_Lac_ads). BPA concentrations in control experiments employing freely suspended, heat-inactivated laccase are also shown (please refer to the Experimental Section for details). Data always represent means ± standard deviations for triplicate experiments.

Irradiated laccase-containing membranes were used to demonstrate the capability of the immobilized biocatalyst to degrade the endocrine disruptor BPA, which was almost completely removed within 48 h (Figure 6). Adsorption or degradation effects originating by the membrane material itself could be excluded because no BPA removal was observed with heat-inactivated, free laccase. Furthermore, sorptive BPA removal observed with enzyme-free membranes did not exceed 15% of the initially applied concentration within 48 h (data not shown). Applying modelling according to first-order kinetics to experiments with laccase-containing membranes, an apparent BPA removal rate of about 20.0 µM/h was obtained (R^2^ > 0.98), corresponding to an apparent specific removal rate (*i.e.*, based on the laccase activity present in the reaction system, expressed as units according to the routine ABTS assay = U; please refer to points 3.5. and 3.6. of the experimental section) of approximately 21.7 µM/h/U. A comparison with the specific BPA removal rate observed with free laccase (about 18.0 µM/h corresponding to 25.3 µM/h/U, R^2^ > 0.97; the time course of BPA concentrations in corresponding experiments is shown in the context of BPA degradation experiments using cryogel-immobilized laccase below; Figure 7) indicates an efficiency of the immobilized enzyme in removing BPA being slightly less of that of the free enzyme. A potential reason could be related to an alteration of the apparent affinity (K_m_ value) of the immobilized biocatalyst for this particular substrate, which may result from a diminished substrate availability due to steric hindrance [45] caused by the E-Beam-generated network of new covalent bonds.

The capability to degrade BPA was also tested with both, cryogels containing E-Beam-immobilized laccase and those containing physically adsorbed laccase (Figure 7). Non-immobilized laccase was used for comparison, and had almost completely removed BPA within 48 h. Control experiments involved heat-inactivated free laccase, where no BPA removal was observed. Sorptive BPA removal observed with enzyme-free cryogel did not exceed 30% of the initially applied concentration within 48 h (data not shown). Upon modelling according to first-order kinetics, apparent BPA removal rates of about 7.4 (R^2^ > 0.99) and 10.4 µM/h (R^2^ > 0.99) were obtained for cryogels containing E-Beam-immobilized (MPC_Lac_EB) and physically adsorbed laccase (MPC_Lac_ads), respectively. These values correspond to apparent specific removal rates (*i.e.*, based on the laccase activity present in the reaction system, expressed as units according to the routine ABTS assay = U) of approximately 61.3 and 43.5 µM/h/U for MPC_Lac_EB and MPC_Lac_ads, respectively. Compared to the apparent specific BPA removal rate of free laccase (derived from the primary solution subsequently used for E-Beam-supported laccase immobilization, Figure 7) of approximately 25.3 µM/h/U as already mentioned before in the context of membrane immobilization approaches, these results suggest an increase in laccase activity and/or stability caused by immobilization as already previously reported [42], which is particularly pronounced in case of the E-Beam-induced enzyme cross-linking. An increase in the activity and affinity for certain substrates of cross-linked laccase was previously observed upon the formation of conjugates with the polymer chitosan, where enzyme hyperactivation by the formation of covalent bonds between laccase and chitosan was suggested [46,47]. With respect to the present work, details regarding the laccase and carrier moieties involved in the E-Beam-induced formation of potentially activity-enhancing new covalent bonds, as well as the nature of such bonds remain to be elucidated. At first glance, random processes would have to be considered for the radical coupling of the enzyme and the carrier as caused by E-Beam irradiation. Clearly higher specific BPA removal rates observed with cryogels than with membranes (see above) regardless of whether E-Beam irradiation was applied or not further may reflect a better availability of BPA to cryogel-immobilized laccase than to membrane-immobilized laccase.

After incubation of laccase-containing irradiated membranes under conditions also applied for BPA degradation experiments (except that BPA was omitted to prevent potential interference; please also refer to point 3.8. of the experimental section for details) for 48 h, 96.1% ± 19.5% (mean ± standard deviation for triplicate experiments) of the initially applied enzyme activity could be recovered from the enzyme-containing membranes. At the same time, only 0.94% ± 0.54% of the initial laccase activity was found to be released into supernatants of corresponding incubation mixtures. In concomitant tests involving freely suspended laccase, 8.1% ± 1.0% of the initial activity could be recovered after 48 h. Altogether these results indicate an efficient prevention from enzyme leakage along with a highly stabilizing effect on laccase activity of the applied membrane immobilization procedure, as also known from other enzyme cross-linking approaches [44,45]. 

Like laccase-containing irradiated membranes, laccase-containing cryogels were evaluated regarding their stability of enzymatic activity. After 48 h of incubation under the operation conditions mentioned before, 57.2% ± 27.3% (mean ± standard deviation for triplicate experiments) of the initial enzyme activity were recovered from cryogels containing E-Beam-immobilized laccase. By contrast, cryogels containing unspecifically adsorbed laccase had lost all of their enzymatic activity after this time. From supernatants of the corresponding incubation mixtures 22.9 ± 24.8 (E-Beam immobilization) and 6.7% ± 5.8% of the initial enzyme activity (laccase adsorption) were recovered. Also considering related effects observed with E-Beam-irradiated membranes, these results clearly indicate:

(i) an activity-stabilizing effect of the E-Beam-induced enzyme cross-linking, as has also been described for other enzyme cross-linking techniques [44,45], and
(ii) that more stable bioreactors with respect to enzymatic activity and leakage from the carrier were obtained with membranes than with cryogels.

**Figure 8:**
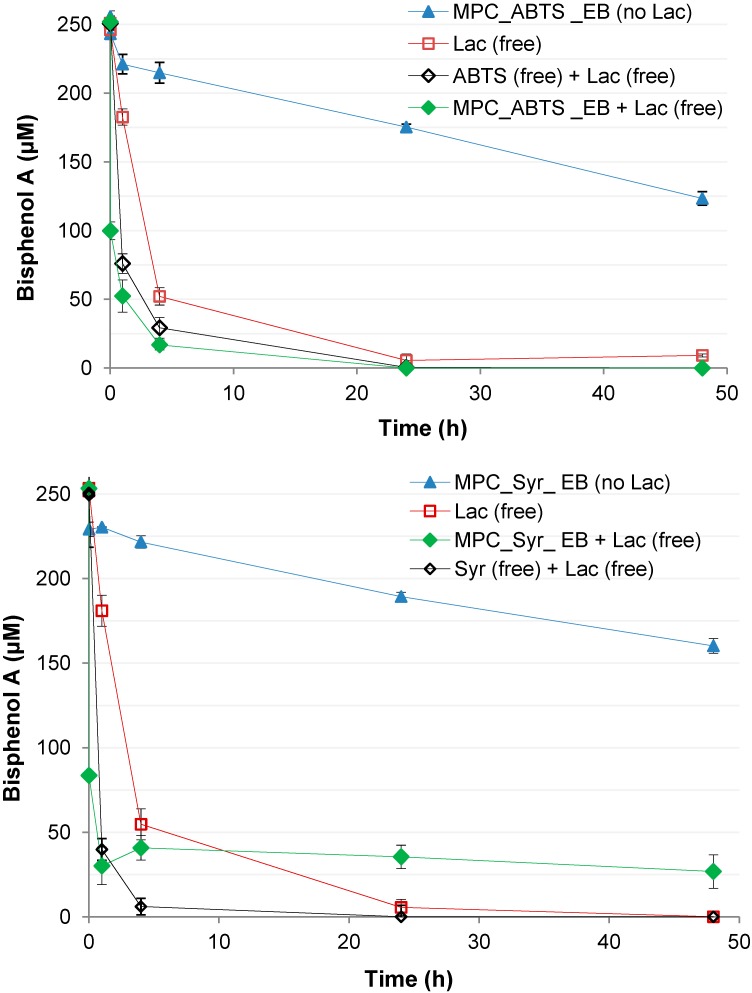
Time courses of BPA concentrations in degradation experiments employing freely suspended laccase (Lac) together with cryogels containing E-Beam-immobilized ABTS (MPC_ABTS_EB, upper figure) and Syr (MPC_Syr_EB, lower figure). Experiments employing freely suspended laccase but no cryogels, and experiments employing ABTS and Syr-supplemented cryogels but no laccase served as controls. Additional experiments involving freely dissolved redox mediators together with free laccase were also conducted for comparison (please refer to the Experimental Section for details). Data always represent means ± standard deviations for triplicate experiments.

Neither the application of membrane-immobilized ABTS nor of syringaldehyde led to a substantial increase in BPA removal rates. This was observed during degradation experiments employing E-Beam immobilization as well as physical adsorption of ABTS and syringaldehyde on membranes together with freely suspended laccase, where control experiments involving BPA and free laccase, but no redox mediator-containing membranes were conducted for comparison (data not shown). By contrast, the presence of freely dissolved redox mediators clearly increased BPA removal catalyzed by free laccase (compare Figure 8). These results may indicate that the redox mediators may have lost their functionality during membrane immobilization, and/or that the immobilized amounts may not have been sufficiently high enough to cause effects. The later assumption is supported by the respective XPS studies (see Section 2.1 and Table 1). Since the E-Beam immobilization is clearly increased when the to-be-immobilized compound is adsorbed to the membrane surface during irradiation, the redox mediators were probably not suitable for this immobilization technique using membranes.

Contrary to the results observed with redox mediator immobilization onto membranes, E-Beam-supported immobilization of ABTS and syringaldehyde in cryogels and the application of such cryogels in degradation experiments additionally involving free laccase resulted in a considerable increase in the initial BPA removal rates, as compared to experiments only employing free laccase but omitting the redox mediator-containing cryogels (Figure 8). As expected, freely dissolved redox mediators used for comparison also clearly increased BPA removal catalyzed by free laccase. Altogether these results clearly indicate that the functionality of the redox mediators was maintained and even improved during cryogel immobilization. This results suggests a different reaction mechanism during the immobilization of redox mediators in cryogels and on membranes. Notably, immobilized redox mediators were found to act more efficiently than in freely dissolved state (especially pronounced for ABTS with a complete BPA removal after 24 h). This could be deduced from a comparison of the time courses of BPA removal in experiments employing free laccase together with immobilized redox mediators, and in those where both laccase and redox mediators were applied in non-immobilized state (Figure 8). The reason might well be related to an accelerated catalytic redox cycle due to the immobilization of redox mediators, potentially preventing their loss from the reaction system. Detailed investigations of such phenomena will be the scope of future work. An increase in the efficiency of the removal of the micro-pollutants BPA, nonylphenol, and triclosan caused by the presence of freely dissolved redox mediators in reaction systems containing immobilized laccase in the form of cross-linked enzyme aggregates (CLEAS) was demonstrated before [48]. However, we are not aware of reports describing the successful application of immobilized redox mediators. It remains to be elucidated inasmuch the immobilization resulted in a stable binding of the redox mediators and whether (and to which extent) they may be released from the cryogels over time.

Also, it needs to be clarified why BPA degradation in reaction mixtures containing cryogel-immobilized syringaldehyde discontinued after a reaction time of 4 h (Figure 8). Furthermore, sorptive binding of BPA to cryogels is indicated by the time course of BPA concentrations in experiments with redox mediator-containing cryogels in the absence of laccase, where redox mediator autoxidation additionally may have contributed to the observed BPA removal especially in case of ABTS (Figure 8). The amounts of incorporated laccase or redox mediators did not exceed 2 wt %, and resulted in highly active enzyme reactors. It is assumed that the costs can be further reduced by decreasing laccase and redox mediator amounts whilst maintaining a high activity.

## 3. Experimental Section

### 3.1. Chemicals and Materials

Poly(vinylidene fluoride) membranes (hydrophobic, pore size 0.45 µm, thickness 125 µm) were purchased from Carl Roth GmbH & Co. (Karlsruhe, Germany). Poly(ethylene glycol) methacrylate (PEGMA, M_n_ = 360 g/mol, purity > 99.0%), tetraethylene glycol diacrylate (TEGDA, purity > 99.5%), Laccase from *Trametes versicolor* (Lac; ≥10 U/mg), syringaldehyde (Syr; purity: 98%), 2,2'-azino-bis(3-ethylbenzothiazoline-6-sulfonic acid) diammonium salt (ABTS; purity ≥ 98%), bisphenol A (BPA; purity > 95%), and phosphate buffered saline (PBS, pH 7.4) were purchased from Sigma-Aldrich (Steinheim, Germany). Bicinchoninic acid (BCA) protein assay reagent A + B was provided by Pierce (Rockford, IL, USA). Tween® 80 for synthesis was obtained from Merck Millipore (Hohenbrunn, Germany). If not otherwise stated Millipore® grade water was used. All chemicals were of analytical grade and used without further purification.

### 3.2. Preparation of Membrane Bioreactors

Prior to enzyme immobilization membrane samples (ϕ 47 mm) were prepared by immersing the membranes for 5 min into ethanol, then the samples were washed 3 × 5 min with water. Now, the membranes were immersed for 5 min in an aqueous buffer solution (PBS, pH 7.0) of laccase at room temperature followed by E-Beam irradiation. We applied doses in the range from 100–200 kGy since previous studies have shown that most radicals are formed on the membrane polymer under these conditions [38,39,40]. 

The control experiment for physical adsorption of laccase at the membrane surface was performed by similar treatment (immersing for 5 min in the laccase solution) without subsequent irradiation. Concentrations and doses are listed in Table 4. Irradiation was performed in an N_2_ atmosphere with O_2_ quantities < 10 ppm using a home-made electron accelerator [49]. The voltage and the current were set to 160 kV and 10 mA, respectively. The absorbed dose was adjusted by the speed of the sample transporter. The modified membrane was rinsed three times per 10 min with buffer solution (referred to as washing solution in the results and discussion section) and afterwards used for further investigations.

**Table 4:** Membrane modification parameters.

Label	Immobilized Compound	Concentration [wt %]	Dose [kGy]
Mem_Ref	-	-	-
Mem_Lac_EB	laccase	0.5	150
Mem_Lac_ads	laccase	0.5	-
Mem_Syr_EB	syringaldehyde	2.0	100
Mem_Syr_ads	syringaldehyde	2.0	-
Mem_ABTS_EB	ABTS	1.0	200
Mem_ABTS_ads	ABTS	1.0	-

### 3.3. Preparation of Cryogel Bioreactors

The synthesis of the cryogels was performed as described previously [26]. The reference cryogel used in the studies was a well-characterized standard cryogel [26,27,28] (MPC_St) synthesized from a solution consisting of 5 wt % PEGMA and 5 wt % TEGDA in PBS, pH 7.4. Briefly, cryogel formulations containing different amounts of laccase, syringaldehyde (treated at 50 °C for 30 min for complete dissolution) and ABTS were weighted in centrifuge tubes according to Table 5, homogenized by gentle shaking and degased by 5 min flushing with nitrogen and 5 min ultrasonification (except for the laccase solutions) at room temperature. 100 mg of each mixture were transferred to 10 mL centrifuge tubes, hermetically sealed, frozen at −20 °C in a cryostat (Lauda, Königshofen, Germany) for 90 min and irradiated with a dose of 12 kGy (supplied in 3 kGy) dose steps by E-Beam of a 10 MeV linear accelerator (Toriy Company, Moscow, Russia) in a home built cooling chamber. It became apparent in previous investigations that a dose of 12 kGy is sufficient for cryogel formation [26]. Further increase of doses did not result in different material properties. Also higher doses could promote polymer degradation. The reaction mixture remains frozen during irradiation. The obtained product is brought to room temperature, transferred to new tubes, and subsequently washed 10× with 250 µL PBS (referred to as washing solution in the results and discussion section). The eluates are collected for the determination of non-bound enzyme or redox mediators. Some cryogels were dried in vacuum at 37 °C for the characterization of the morphology and chemical composition.

**Table 5:** Composition of the cryogel mixtures (in PBS buffer).

	Laccase/Syringaldehyde/ABTS (wt %)	Standard Formulation (wt %)
**MPC_St**	-	100
**MPC_Lac**	0.25	99.75
**MPC_Syr**	0.50	99.50
**MPC_ABTS**	2.00	98.00

In order to determine the amount of non-specific adsorbed (e.g., by electrostatic or hydrophobic interactions) laccase or redoxmediators in each case 5 standard cryogels were incubated for 30 min with 90 mg of 0.25 wt % laccase, 0.5 wt % syringaldehyde and 2 wt % ABTS in PBS followed by 10x times washing with 250 µLPBS (MPC_Lac_ads; MPC_Syr_ads; MPC_ ABTS_ads).

### 3.4. Polymer Characterization

The morphology of the porous materials was studied by scanning electron microscopy (SEM, Ultra 55, Carl Zeiss SMT, Jena, Germany). In order to prevent charging the sample was sputtered with a thin gold layer. Chemical composition was analyzed with X-ray photoelectron spectroscopy (AXIS Ultra, Kratos Analytical, Manchester, England). The kinetic energy of the electrons was analyzed with a pass energy of 160 eV for the survey spectra and 40 eV for the energy resolved spectra, respectively. Thermal stability of the MPCs was examined by thermogravimetric analysis (TGA 7, Perkin Elmer, Waltham, MA, USA).The reaction yield (gel content) was determined as follows: the freshly prepared and washed cryogels were dried in vacuum (T = 37 °C) to constant mass. The gel content GC was calculated using Equation (1):

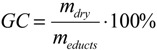
(1)
where *m_dry_* is the mass of the final dried cryogels and *m_educts_* the mass of the educts. The equilibrium swelling degree SD of the standard cryogels was determined after 30 min swelling in PBS. Access water was removed by wiping on a wet tissue paper, weighted (*m_swollen_*) and the SD calculated as follows:

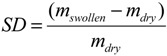
(2)


In all cases at least three independent samples were investigated. Laccase concentrations on the membranes were investigated using the bicinchoninic acid kit [50]. The beforehand modified membranes, stored in the buffer solution were shaken for 1 h at room temperature. Afterwards, the samples were washed three times with 1 mL of PBS buffer solution (pH 7.0). Then, the BCA reagent was added to the membrane samples and the plate was incubated for 25 min at 37 °C. The plate was then shaken for 5 min at room temperature, the solution was transferred to a new microtiter plate and light adsorption at 562 nm was measured using a microtiter plate reader (Infinite M200, Tecan, Crailsheim, Germany). For calibration, seven laccase concentrations of 5.00, 2.50, 1.25, 0.63, 0.31, 0.16 and 0.00 µg/mL were used.

### 3.5. Laccase Activity Assays

The activity of freely suspended laccase in primary and washing solutions was determined by spectrophotometry by monitoring the oxidation of 2 mM 2,2'-azino-bis(3-ethylbenzthiazoline-6-sulfonic acid) (ABTS) at pH 4.0, using a microplate reader operated at 420 nm as previously described [51]. One unit of laccase activity corresponds to 1 μmol product formed per min. For activity determination of laccase immobilized by E-Beam treatment and by physical adsorption on membranes and cryogels, a discontinuous assay based on recording of the oxidation of ABTS was applied. Membranes and cryogels containing immobilized laccase were incubated in 10 mL of McIlvaine buffer [52] (pH 4.0) additionally containing 2 mM ABTS, under agitation at 120 r.p.m. The enzymatic ABTS oxidation was determined by taking an aliquot sample from the supernatant every minute, and recording the absorbance at 420 nm with a microplate reader as mentioned before. Data points yielding maximal slopes of ABTS oxidation were used to calculate the enzyme activity.

### 3.6. Degradation of BPA by Immobilized Laccase

All degradation experiments of BPA were conducted in 22-mL clear glass vials. The corresponding reaction mixtures always contained 250 µM BPA in 4 mL McIlvaine buffer (pH 4.0). BPA was aseptically added from a 25 mM stock solution in methanol containing 10% (m/v) Tween 80 in addition to improve the solubility, yielding a final concentration of 250 μM BPA (corresponding to 1% [v/v] methanol and 0.1% [m/v] Tween 80) [53] Immobilized and freely suspended laccase were included in degradation experiments as described in more detail below. Incubation was always carried out at room temperature (22 ± 2 °C) and agitation at 120 r.p.m. for 48 h. Samples were taken before adding the respective enzyme preparation, and at the time points indicated in the text. Degradation experiments employing laccase-containing irradiated membranes contained 0.92 U immobilized laccase (Mem_Lac_EB in Figure 6). Controls were prepared by firstly heat-inactivating 0.95 U freely suspended laccase for 1 h at 95 °C, which was then applied in corresponding reaction mixtures.

Degradation experiments with cryogels containing E-Beam-immobilized (MPC_Lac_EB in Figure 7) and unspecifically adsorbed laccase (MPC_Lac_ads in Figure 7) involved 0.12 and 0.24 U immobilized laccase, respectively. In corresponding experiments with freely suspended laccase, 0.71 U laccase derived from the primary solution subsequently used for E-Beam-supported immobilization was applied. Heat-inactivated controls were prepared from 0.475 U freely suspended laccase, under conditions already described above.

For calculation of apparent BPA removal rates as based on application of first-order kinetics, the exponential fitting function of Microsoft Excel (2010) was applied to BPA concentration *vs.* time plots. The apparent first-order rate constants for BPA removal and the initial BPA concentrations thus obtained were used to calculate the apparent BPA removal rates.

### 3.7. BPA Removal in the Presence of Immobilized Redox Mediators

BPA degradation experiments employing redox mediators were carried out under the conditions already described above (Section 3.6.), with the following modifications: In experiments involving freely suspended laccase together with immobilized redox mediators, laccase was included at 0.95 U, and redox mediators were applied as follows: 8.4 mg (dry mass) of cryogel containing E-Beam-immobilized syringaldehyde (corresponding to a 2% syringaldehyde solution used for cryogel production), 8.4 mg (dry mass) of cryogel containing E-Beam-immobilized ABTS (corresponding to a 2% ABTS solution used for cryogel formation). In experiments employing freely suspended laccase (applied as above) and freely dissolved redox mediators, the latter (derived from primary solutions subsequently used for the production of redox mediator-containing cryogels) were applied at 180 µM (ABTS) and 545 µM (syringaldehyde). Additional experiments involved free laccase (applied as above) only, and cryogel-immobilized redox mediators (amounts as above) but omitting laccase.

### 3.8. Stability Testing of the Immobilized Laccase

In order to evaluate the catalytic stability, immobilized and freely suspended laccase was incubated under the conditions already described in Section 3.6. (except that BPA was omitted). Stability tests employing laccase-containing irradiated membranes initially contained 0.13 U immobilized laccase. Stability tests with cryogels containing E-Beam-immobilized and unspecifically adsorbed laccase initially involved 0.12 and 0.10 U immobilized laccase, respectively. Freely suspended laccase was initially applied at 0.11 U in additional tests. The enzyme activity as determined after 48 h was compared with that initially applied, respectively. For activity determinations the routine ABTS assay described in Section 3.5. was applied to both immobilized laccase and supernatants of the corresponding incubation mixtures, in order to monitor the potential enzyme release from membranes/cryogels.

### 3.9. Analysis of BPA by Ultra Performance Liquid Chromatography (UPLC)

Aqueous samples (0.5 mL) were placed in 1.5-mL Eppendorf tubes, supplemented with 0.5 mL methanol, thoroughly mixed, and stored at −20 °C until further use. Before analysis, samples were centrifuged at 14.000 rpm and 4 °C for 15 min (Eppendorf centrifuge 5804R; rotor type 16 F24-11; Eppendorf, Hamburg, Germany). After centrifugation, supernatants (900 μL) were transferred into 1.5‑mL HPLC/UHPLC vials, which were then tightly closed with screw caps containing silicone/PTFE septa. Aliquots from samples (3.3 μL) were directly subjected to an Acqutity^TM^ UPLC system comprising of a Binary Solvent Manager (BSM), a Sample Manager (SM), and a PDA eλ photo diode array detector, and equipped with an Acquity^TM^ UPLC BEH C18 column (1.7 μm particle size; 2.1 × 50 mm; Waters, Eschborn, Germany) operated at a column temperature of 40 °C. The following solvents served as mobile phases: solvent A - 10% methanol (gradient grade, Th. Geyer, Renningen, Germany) in deionized water (Q-Gard 2, Millipore, Schwalbach, Germany); solvent B - 100% methanol. The following elution profile was applied: isocratic elution at 20% B for 0.14 min; linear increase to 100% B until 2.8 min; isocratic elution at 100% B until 3.2 min; linear decrease to 20% B until 3.25 min; isocratic elution at 20% B until 3.5 min (0.5 mL/min flow rate). A wavelength range from 220 to 400 nm was recorded (detection wavelength: 278 nm). Calibration of the method was carried out using external BPA standards.

## 4. Conclusions

Novel porous membrane and cryogel-based bioreactors with the covalently immobilized enzyme laccase, and the two redox mediators 2,2'-azino-bis(3-ethylbenzothiazoline-6-sulphonic acid) and syringaldehyde were successfully accomplished using E-Beam irradiation in a water-based one-step approach. It was demonstrated that the resulting bioreactors possess high enzymatic activity and efficiently degraded bisphenol A as model pollutant to completion in less than 48 h. Furthermore, we studied for the first time the immobilization of redox mediators which resulted in bioreactors with higher activity compared the non-immobilized approaches. The increased surface area of the porous structure enhances the accessibility of the reactive binding sites of the immobilized laccase or redox mediators. This new immobilization technique provides a directed, fast, and environmentally friendly method for enzyme immobilization on various polymer substrates and allows for the repeated use of the bioreactor.
